# The effects of daylight saving time clock changes on accelerometer‐measured sleep duration in the UK Biobank

**DOI:** 10.1111/jsr.14335

**Published:** 2024-10-21

**Authors:** Melanie A. de Lange, Rebecca C. Richmond, Kate Birnie, Chin Yang Shapland, Kate Tilling, Neil M. Davies

**Affiliations:** ^1^ MRC Integrative Epidemiology Unit University of Bristol Bristol UK; ^2^ Population Health Sciences, Bristol Medical School University of Bristol Bristol UK; ^3^ NIHR Oxford Health Biomedical Research Centre University of Oxford Oxford UK; ^4^ Division of Psychiatry University College London London UK; ^5^ Department of Statistical Science University College London London UK; ^6^ K.G. Jebsen Center for Genetic Epidemiology, Department of Public Health and Nursing Norwegian University of Science and Technology Trondheim Norway

**Keywords:** actigraphy, daylight saving time, sleep, sleep loss, UK Biobank

## Abstract

We explored the effects of daylight saving time clock changes on sleep duration in a large accelerometer dataset. Our sample included UK Biobank participants (*n* = 11,780; aged 43–78 years) with accelerometer data for one or more days during the 2 weeks surrounding the Spring and Autumn daylight saving time transitions from October 2013 and November 2015. Between‐individual *t*‐tests compared sleep duration on the Sunday (midnight to midnight) of the clock changes with the Sunday before and the Sunday after. We also compared sleep duration on all other days (Monday–Saturday) before and after the clock changes. In Spring, mean sleep duration was 65 min lower on the Sunday of the clock changes than the Sunday before (95% confidence interval −72 to −58 min), and 61 min lower than the Sunday after (95% confidence interval −69 to −53). In Autumn, the mean sleep duration on the Sunday of the clock changes was 33 min higher than the Sunday before (95% confidence interval 27–39 min), and 38 min higher than the Sunday after (95% confidence interval 32–43 min). There was some evidence of catch‐up sleep after both transitions, with sleep duration a little higher on the Monday–Friday than before, although this was less pronounced in Autumn. Future research should use large datasets with longer periods of accelerometer wear to capture sleep duration before and after the transition in the same individuals, and examine other aspects of sleep such as circadian misalignment, sleep fragmentation or daytime napping.

## 1 INTRODUCTION

Daylight saving time (DST), the practice of moving the clocks 1 hr forward in Spring and 1 hr back in Autumn, was introduced in World War One to maximise exposure to daylight during the working day and reduce energy use (Malow, 2022). It operates in about 70 countries, including the UK, and affects over a quarter of the world's population (Hansen et al., 2017; Zhang et al., 2020). However, growing evidence suggests that the DST clock changes may adversely affect population health. For example, studies have found an increased incidence of myocardial infarction (Jiddou et al., 2013), atrial fibrillation (Chudow et al., 2020), strokes (Sipilä et al., 2016) and fatal traffic accidents (Fritz et al., 2020) in the weeks immediately following the Spring clock change, as well as an increased risk of depressive episodes after the Autumn transition (Hansen et al., 2017). These health consequences are thought to be mediated by sleep deprivation and circadian disruption (Fritz et al., 2020; Hansen et al., 2017; Lim et al., 2023; Sipilä et al., 2016).

A review of DST sleep studies by Harrison (2013) reported that both Spring and Autumn clock changes were associated with less sleep for about a week after the change (Harrison, 2013a). Sleep after the Spring change was found to be more fragmented, and people took longer to get to sleep, whilst sleep loss after the Autumn change was due to people continuing to wake early (Harrison, 2013a). However, estimates of the exact effects of clock changes on sleep duration do vary. A recent analysis of subscribers to a fitness tracker platform in the USA (75% male, mean age 38 years) reported that sleep duration on the night of the Spring clock change was 5 min less than the average of the same night on surrounding weekends (Heacock et al., 2022), whilst analysis of self‐report data from older participants in a USA cohort study (mean age 61 years, 60% female) found that people slept for 30 min less on the night of the Spring clock change than the previous weekend (Owen et al., 2022), and an examination of participants aged ≥ 15 years in the American Time Use Survey reported that on average people slept 42 min less on the night of the Spring clock change than the weekend before and after (Michelson, 2011). Meanwhile, estimates of sleep duration on the night of the Autumn clock change range from no difference (92 study participants wearing a headband sleep monitor; Shambroom & Fabregas, 2010) to 20 min more (subscribers to fitness tracker) (Heacock et al., 2022) or 40 min more (American Time Use Survey) (Michelson, 2011) than on surrounding weekends.

Estimates of sleep duration on the weekdays after the clock changes also differ considerably. One study of 10 participants wearing wrist‐worn accelerometers (aged 32–70 years, 60% female) reported that sleep duration on the Monday–Wednesday nights after the Spring change was 60 min less than the previous week (Lahti et al., 2006a). This reduction in sleep is supported by analysis of working adults (mean age 42 years, 51% female) from the American Time Use Survey that showed a 40‐min reduction in sleep on the Monday after the Spring transition (Barnes & Wagner, 2009). In contrast, an analysis of actigraphy data from 14 male students (mean age 27 years) found that sleep duration was 28 min higher on the Monday–Friday after the Spring clock change than before (Tonetti et al., 2013). Other estimates fall within these extremes, with the majority showing a reduction in weekday sleep after the Spring transition (Medina et al., 2015; Michelson, 2011; Völker et al., 2023; Zolfaghari et al., 2023).

Estimates of sleep duration on the weekdays after the Autumn changes are also conflicting. Most studies have found little difference in sleep duration on the weekdays before and after the Autumn transition (Barnes & Wagner, 2009; Michelson, 2011; Zolfaghari et al., 2023). However, one study of male students reported a 25‐min reduction in sleep on the weekdays following the clock change (Tonetti et al., 2013). Conversely, an analysis of survey data from adults (aged > 18 years) in the US Behavioural Risk Factor Surveillance System reported sleep duration being 2 min higher per night in the week after the Autumn transition than before (Jin & Ziebarth, 2020).

Potential modifiers of the effect of the clock changes on sleep duration include chronotype (morning/evening preference) and habitual sleep duration. The Spring change is thought to have the most detrimental effect on the sleep duration of evening types and short sleepers (< 8 hr), whilst morning types and long sleepers (> 8 hr) lose the most sleep in Autumn (Harrison, 2013b; Kantermann et al., 2007; Lahti et al., 2006b; Lahti et al., 2008; Tyler et al., 2021). In addition, the negative impact of the Spring clock change on sleep efficiency (proportion of time in bed spent asleep) has been found to be more pronounced in those with a prior sleep debt (not meeting your preferred sleep duration) in the week before the clock change (Lahti et al., 2006a).

Many studies of the effects of DST transitions on sleep have relied on subjective self‐report data (Barnes & Wagner, 2009; Jin & Ziebarth, 2020; Michelson, 2011; Owen et al., 2022; Völker et al., 2023; Zolfaghari et al., 2023), which may be affected by recall bias, and have been shown to only moderately correspond to more objective measures of sleep, such as actigraphy (Lauderdale et al., 2008). Whilst some DST research has used actigraphy data, most of these studies have suffered from small sample sizes (*n* < 100) (Lahti et al., 2006a; Medina et al., 2015; Shambroom & Fabregas, 2010; Tonetti et al., 2013), reducing their estimates' precision. In this study, we used accelerometer data from the UK Biobank to explore the effects of the DST clock changes on sleep duration in a much larger sample (*n* = 11,780).

## 2 METHODS

### 2.1 Study population

**FIGURE 1:**
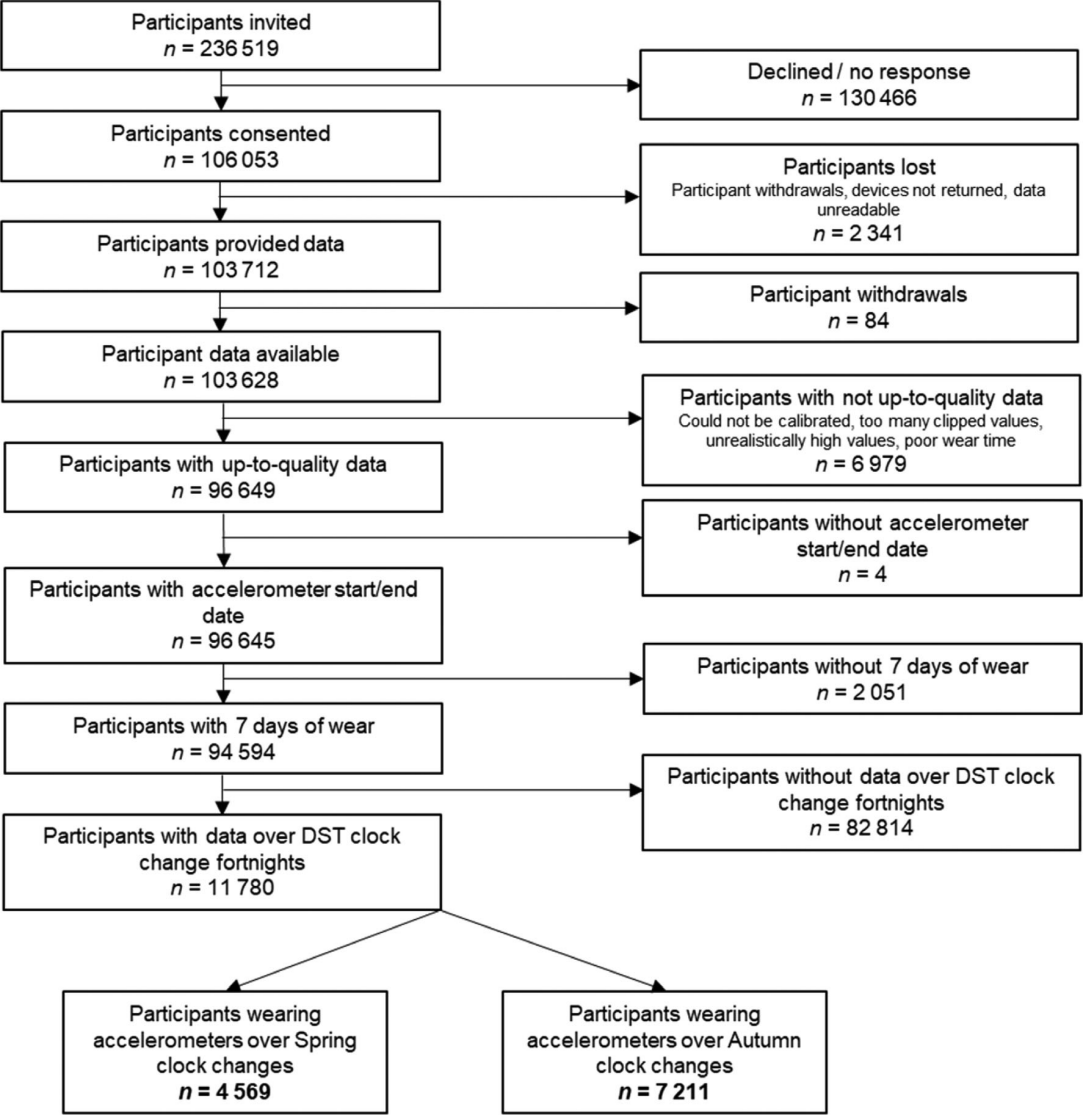
Participant flow diagram.

The UK Biobank is a prospective cohort study with data on about 500,000 people aged 40–69 years when recruited from across the UK in 2006–2010 (Sudlow et al., 2015). The participantion rate was 5.5% (Fry et al., 2017). At baseline, participants completed questionnaires covering sociodemographic and lifestyle factors, and their medical history. Participants also had physical (e.g. blood pressure) and biological (e.g. blood/urine samples) measures taken (Sudlow et al., 2015). 236,519 participants who had provided an email address were randomly selected and invited to wear an accelerometer for 7 days between June 2013 and December 2015. Of these, 106,053 agreed (participation rate 44.8%) (Doherty et al., 2017), and 103,712 ultimately provided accelerometer data. After subsequent participant withdrawals, accelerometer data were available for 103,628 participants (Figure 1).

In keeping with previous analyses (Doherty et al., 2017; Doherty et al., 2018), for quality control, we excluded participants if they had poor wear time (failing to have ≥ 3 days of data and data in each hour of the 24‐hr day over multiple days), poor calibration (insufficient data preventing recalibration by the previous or next measurement), unrealistically high activity levels (an average overall volume of activity per day of over 100 milli‐gravity units [mg]), or if over 1% of values were clipped before or after calibration (a clipped value is when the measured value exceeds the maximum or minimum values the device can measure and therefore any excess acceleration is not recorded). We also excluded records of those without accelerometer wear start and end dates, and those with less than 7 days of wear.

In this study, participants wore Axivity AX3 triaxial accelerometers on their dominant wrist (described in detail elsewhere) (Doherty et al., 2017). Participants were asked to wear the accelerometer continuously and complete their daily activities as normal (Doherty et al., 2017). They wore the accelerometers for 168 hr (7 × 24 hr), starting at 10:00 hours on day 1, and finishing at 10:00 hours on day 8. Consequently, day 1 data were made up of data from 10:00 hours to 24:00 hours on day 1, combined with data from 24:00 hours to 10:00 hours on day 8. For our study, this meant we had to exclude day 1 for all participants as it consisted of data from more than 1 day. We therefore only included participants who had data (excluding their day 1) for any of the days during the 2 weeks surrounding the DST clock changes (the 15 days from the Sunday before the clock change to the Sunday after). This resulted in a final sample of 11,780 participants, 4569 in the Spring and 7211 in the Autumn (Figure 1). Our study covered two Spring clock changes (2014 and 2015) and three Autumn clock changes (2013, 2014 and 2015).

UK Biobank participants started wearing their accelerometers on different days of the week, with no participants starting on a Tuesday or Sunday. In our final sample, participants started to wear their accelerometer from two Mondays before the clock changes to the Saturday after the clock changes. Having excluded data from the first day for each participant, our resulting dataset included data from the Sunday before the clock changed to the Sunday after the clock changed, with different numbers of participants per day (Table S1). No participants had data for the whole study period.

### 2.2 Accelerometer‐measured sleep duration

Sleep duration has been derived from the raw UK Biobank accelerometer data by Doherty et al. (2017). This includes the proportion of time (from midnight to midnight) spent asleep overall, by day, on weekends and weekdays, and broken down by hour (overall, on weekends and weekdays). Doherty et al. adjusted the data of those wearing the accelerometers over the clock changes so that the Sundays of the change comprised 24 hr (instead of 23 or 25 hr). In Spring (when the clocks go forward an hour at 01:00 hours and we lose an hour), they imputed an hour of data for the hour missing between 01:00 hours and 02:00 hours. In Autumn (when the clocks go back an hour at 02:00 hours and we gain an hour), they overwrote the data for the hour between 01:00 hours and 02:00 hours (effectively deleting an hour of data).

To accurately capture the effects of the clock changes in this study, we adjusted the sleep proportion on the Sunday of the clock changes to add/subtract the hour deleted/imputed by Doherty et al. The sleep proportion for the hour that we added/subtracted was the average sleep proportion between 01:00 hours and 02:00 hours for the 96,645 UK Biobank participants who wore accelerometers on normal weekends (0.90). In Spring, our sleep proportion on the Sunday of the clock changes was calculated by the following formula: (Doherty sleep proportion on Sunday × 24–0.90)/23. In Autumn, the following formula was applied: (Doherty sleep proportion on Sunday × 24 + 0.90)/25. We then rescaled the sleep proportion data to duration in minutes by multiplying data for Monday–Saturday and normal Sundays by 1440 (the number of minutes in 24 hr). Data for the Sundays of the clock changes were multiplied by 1380 in Spring (to adjust for the Sunday only having 23 hr) and by 1500 in Autumn (to adjust for the Sunday having 25 hr). For examples of these adjustments, see Data S1.

### 2.3 Sociodemographic characteristics

Several sociodemographic characteristics were considered potential effect modifiers of the effect of the clock changes on sleep duration. Sex (male or female) was obtained from a central registry at recruitment, but sometimes updated by the participant. Age (43–58 years, 59–67 years or 68–78 years) was calculated using age at recruitment and date of accelerometer wear. Other potential effect modifiers were self‐reported current employment status (employed, retired or other), self‐reported habitual sleep duration in whole hours (≤ 6 hr, 7–8 hr or ≥ 9 hr) and self‐reported chronotype (morning, no preference or evening). Self‐reported habitual sleep duration was only used for sample characterisation purposes. Our main analysis explores the effects of the clock changes on accelerometer‐measured sleep duration. Detailed information on how the sociodemographic variables were derived is provided in Data S1.

### 2.4 Statistical analysis

Mean daily sleep duration (from midnight to midnight) for the 2 weeks surrounding the Spring and Autumn clock changes was plotted (see Table S1 for sample sizes per day). We conducted between‐individual *t*‐tests to compare participants' sleep duration on the Sunday of the clock changes with the Sunday before and the Sunday after the transitions. We also conducted between‐individual *t*‐tests to compare sleep duration on all other days (Monday–Saturday) before and after the clock changes. To establish whether sociodemographic factors modified the effects of the clock changes, all analyses were then stratified separately by age, sex, current employment status, self‐reported habitual sleep duration, and chronotype. Cochran's Q was used to test heterogeneity between strata. It was impossible to conduct within‐person comparisons of sleep duration on the Sunday of the clock change to the Sunday before and after, or the same weekdays before and after, because participants only had 6 days of usable accelerometer data each. To see whether the presence of nightshift workers in our main sample affected our results, we conducted a sensitivity analysis in which we re‐ran our analysis with these participants excluded.

Analyses were performed in Stata version 16 via JupyterLab in DNA Nexus. The full code for data cleaning and analysis is available at https://github.com/MeldeLange/dst_accel. This research was pre‐specified as part of UK Biobank application 86626.

## 3 RESULTS

### 3.1 Participant characteristics

In this study, 44% of participants were male, 66% were aged 59 years and over, and 32% were retired (Table 1). In our sample, 72% reported sleeping for 7–8 hr per night, and 57% stated they were a morning chronotype.

**TABLE 1:** Sample characteristics.

	Total sample	Spring clock change	Autumn clock change
Total (*n*)	11,780	4569	7211
Variables *n* (%)			
Sex			
Male	5165 (43.8%)	1975 (43.2%)	3190 (44.2%)
Age (years)			
Tertile 1 (age 43–58)	3988 (33.9%)	1599 (35.0%)	2389 (33.1%)
Tertile 2 (age 59–67)	4486 (38.1%)	1707 (37.4%)	2779 (38.5%)
Tertile 3 (age 68–78)	3306 (28.1%)	1263 (27.6%)	2043 (28.3%)
Current employment status			
Paid employment/self‐employed	7141 (60.8%)	2815 (61.8%)	4326 (60.1%)
Retired	3769 (32.1%)	1426 (31.3%)	2343 (32.6%)
Other	842 (7.2%)	313 (6.9%)	529 (7.3%)
Sleep duration (hr) (self‐reported)			
≤ 6 hr	2519 (21.5%)	999 (21.9%)	1520 (21.2%)
7–8 hr	8467 (72.2%)	3270 (71.8%)	5197 (72.4%)
9 or more hr	748 (6.4%)	284 (6.2%)	464 (6.5%)
Chronotype (self‐reported)			
Morning	6707 (57.4%)	2558 (56.4%)	4149 (58.0%)
No preference	1209 (10.3%)	473 (10.4%)	736 (10.3%)
Evening	3777 (32.3%)	1507 (33.2%)	2270 (31.7%)

### 3.2 Mean sleep duration by day

**FIGURE 2:**
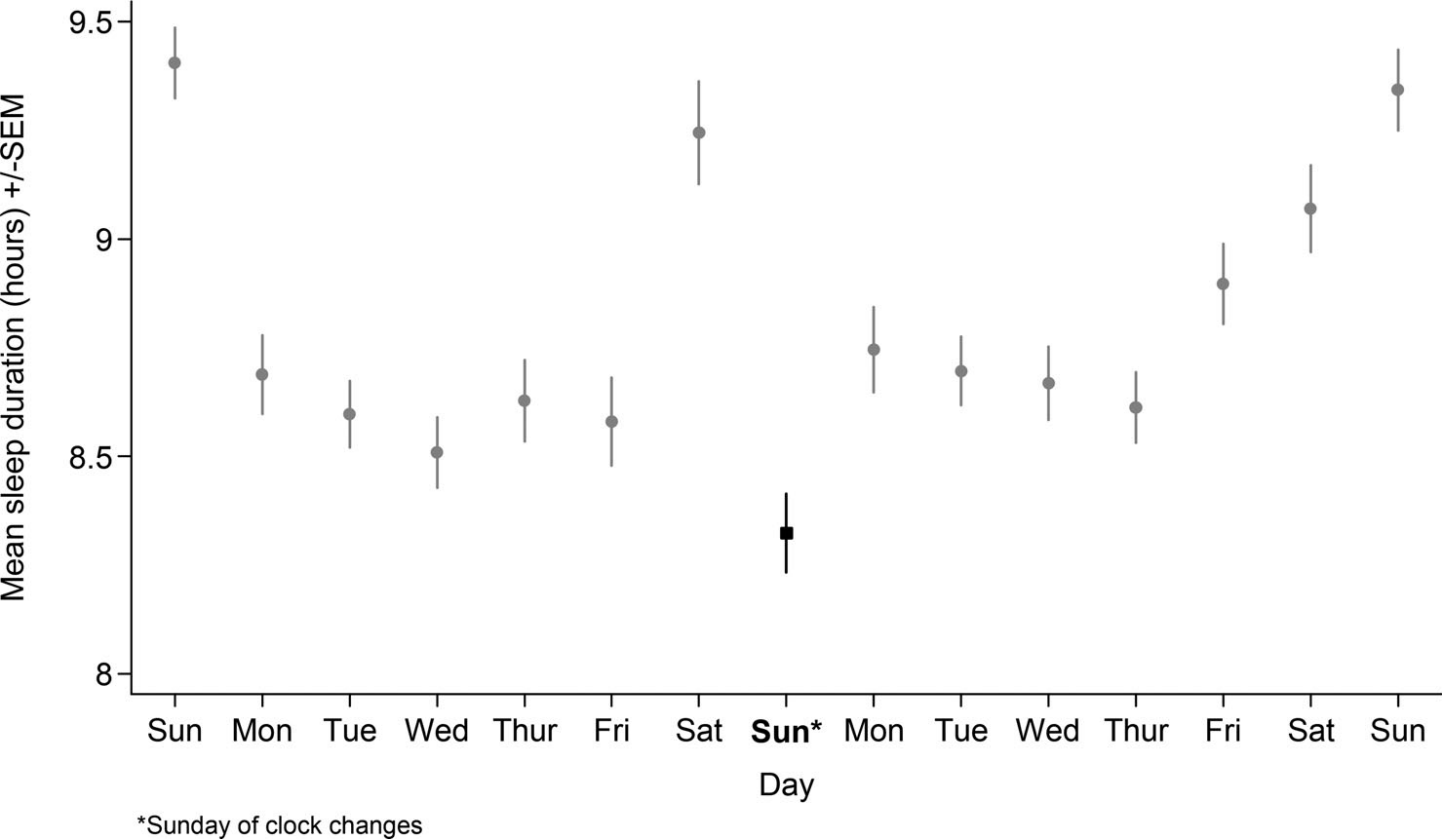
Mean daily sleep duration by day over the Spring clock changes. Dot/square shows the mean. Bar shows the standard error of the mean.

**FIGURE 3:**
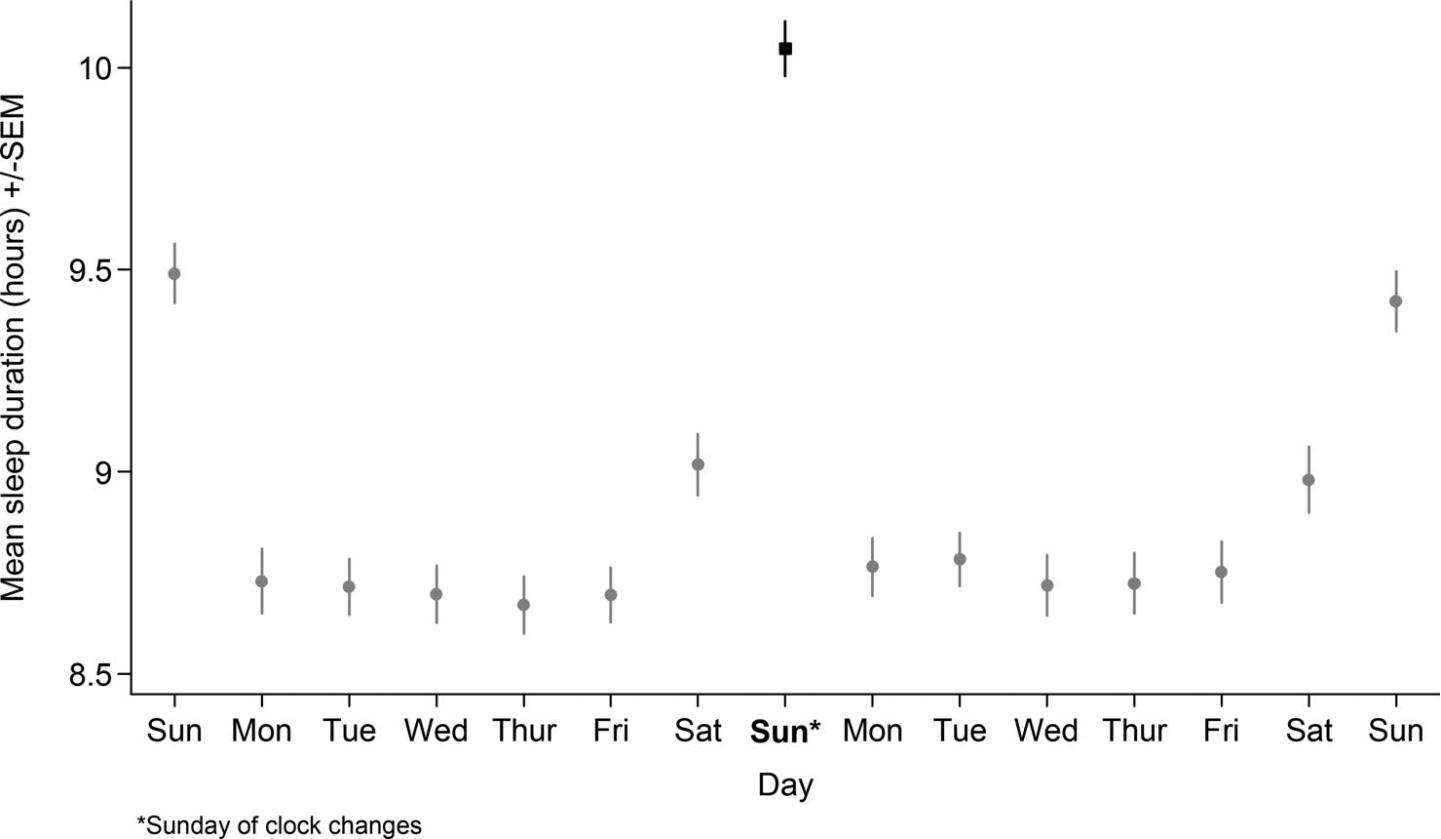
Mean daily sleep duration by day over the Autumn clock changes. Dot/square shows the mean. Bar shows the standard error of the mean.

Figures 2 and 3 show mean sleep duration by day over the fortnight surrounding the Spring and Autumn clock changes (see Table S1 for sample sizes per day, and Table S2 and Figures S1–S10 for stratified results). Sleep duration on the Sunday of the clock changes was lower (in Spring) and higher (in Autumn) than on all other days, including the previous and subsequent Sundays. In general, sleep duration tended to be a little higher on the Monday–Friday after both clock changes than before, although this difference was less pronounced in Autumn.

### 3.3 Between‐individual comparisons of sleep duration on the Sunday of the clock change versus the Sunday before and the Sunday after

Between‐individual *t*‐tests comparing people's sleep duration on the Sunday of the clock changes with the Sunday before and the Sunday after are shown in Table 2 (see Table S3 for stratified results). In Spring, mean sleep duration on the Sunday of the clock changes (8 hr 19 min) was 65 min lower than the Sunday before (95% confidence interval [CI] −72 to −58 min) and 61 min lower than the Sunday after (95% CI −69 to −53).

**TABLE 2:** Between‐individual comparisons of mean sleep duration (hr and min) on the Sunday the clock changes versus the Sunday before and the Sunday after.

	Sunday clock change		Sunday before				Sunday after			
	*n*	Mean sleep duration (SD)	*n*	Mean sleep duration (SD)	Difference in min (SE)	*p*‐Value*	*n*	Mean sleep duration (SD)	Difference in min (SE)	*p‐*Value*
Spring	1515	8 hr 19 min (1 hr 48 min)	1785	9 hr 24 min (1 hr 45 min)	−64.87 (3.72)	3.667 × 10^−65^	1269	9 hr 21 min (1 hr 41 min)	−61.14 (4.00)	9.902 × 10^−51^
Autumn	2688	10 hr 3 min (1 hr 47 min)	2277	9 hr 29 min (1 hr 46 min)	33.39 (3.04)	8.065 × 10^−28^	2246	9 hr 25 min (1 hr 46 min)	37.51 (3.05)	2.677 × 10^−34^

SD, standard deviation; SE, standard error.

* *p*‐Value from *t*‐tests.

In Autumn, mean sleep duration on the Sunday of the clock changes (10 hr 3 min) was 33 min higher than the Sunday before (95% CI 27–39 min) and 38 min higher than the Sunday after (95% CI 32–43 min). There was little evidence of differences by sociodemographic characteristics for either transition, with lower sleep on the Sunday of the Spring transition and higher sleep on the Sunday of the Autumn transition seen for all sociodemographic subgroups (see Table S3 for stratified results and Cochran's Q test of heterogeneity results).

### 3.4 Between‐individual comparisons of sleep duration on Monday–Saturday after the clock change versus the same day the week before

Table 3 highlights in greater detail the trend over both Spring and Autumn transitions of sleep duration being higher on the Monday–Friday after the clock changes than before, as well as being lower on the Saturday after the clock change than the Saturday before. However, as already mentioned, this pattern was much less marked in Autumn. The mean difference in sleep duration between weekdays (Monday–Friday) after and before the Spring clock change was 7.4 min, whereas in Autumn the average weekday difference was 2.9 min. We detected differences by sex, with women generally tending to sleep for fewer minutes on weekdays after the Spring clock change than before, whilst men generally slept for longer on weekdays after the Spring clock change than before. This pattern (albeit on a smaller scale) was also seen after the Autumn change. There was no obvious pattern to the changes after the Spring clock changes across age, self‐reported sleep duration, employment or chronotype groups. After the Autumn clock change, a general pattern was that the youngest tertile exhibited a positive change (slept longer after the clock change), the middle tertile had a smaller positive or negative change, and the oldest tertile tended to have a negative change. In addition, retired people tended to sleep less on the weekdays after the Autumn clock change than before, whilst the opposite was true for those who were employed (see Figures S1–S5 and Table S4 for stratified results and Cochran's Q test of heterogeneity).

**TABLE 3:** Between‐individual comparisons of mean sleep duration (hr and min) on the Monday–Saturday before and after the Spring and Autumn clock changes.

Spring					Autumn			
	*n*	Difference in sleep duration (min)	95% CI	*p‐*Value	*n*	Difference in sleep duration (min)	95% CI	*p‐*Value
Mon after versus before	2518	3.45	−4.52, 11.42	0.396	3839	2.17	−4.26, 8.61	0.508
Tue after versus before	3646	5.93	−0.65, 12.50	0.077	4935	4.12	−1.57, 9.81	0.156
Wed after versus before	3018	9.53	2.51, 16.56	0.008	4080	1.36	−4.81, 7.53	0.666
Thur after versus before	2906	−0.95	−8.37, 6.48	0.803	3945	3.26	−2.94, 9.46	0.303
Fri after versus before	2491	19.01	10.80, 27.22	5.945 × 10^−06^	4309	3.38	−2.68, 9.45	0.274
Sat after versus before	2043	−10.46	−19.76, −1.16	0.028	3857	−2.28	−8.94, 4.38	0.502

CI, confidence interval.

### 3.5 Sensitivity analysis

Our main analysis included 465 participants who self‐reported doing nightshift work. This represented 4.8% of our total sample. Our sensitivity analysis, in which we excluded these nightshift workers, included 11,315 participants: 4377 in Spring and 6938 in Autumn (see Tables S5 and S6 for sensitivity analysis sample sizes per day and sample characteristics). Figures S11 and S12, and Tables S7 and S8 show that the results of our sensitivity analyses were highly consistent with our main results.

## 4 DISCUSSION

In this study, we used accelerometer data from 11,780 UK Biobank participants to examine the effects of the DST clock changes on sleep duration. In the Spring, our data showed that the mean sleep duration was 65 min lower on the Sunday of the clock changes than on the Sunday before, and 61 min lower than on the Sunday after. These results suggest that the Spring transition is associated with reduced sleep duration. It is difficult to directly compare our results that look at sleep duration each day (from midnight to midnight) with studies that examine sleep each night (from noon to noon) (Heacock et al., 2022; Owen et al., 2022). However, we found a loss of sleep greater than that seen in previous studies, which have reported a 5–30‐min reduction in sleep duration on the night of the Spring clock change (Heacock et al., 2022; Owen et al., 2022) and a study reporting a 42‐min reduction on the Sunday of the change (Michelson, 2011).

In Autumn, we found that mean sleep duration was 33 min higher on the Sunday of the clock changes than the Sunday before, and 38 min higher than on the Sunday after. This suggests that the Autumn transition is associated with increased sleep duration. However, participants did not (or could not) take advantage of the full extra hour of increased sleep. This may be because their body clocks or social commitments prevent them from having an entire hour more sleep. Our results are consistent with the existing literature, with one earlier study reporting a 20‐min increase on the night of the Autumn clock change compared with surrounding weekends (Heacock et al., 2022), and another reporting a 40‐min increase in sleep on the Sunday of the change (Michelson, 2011).

We found that sleep duration was higher on the weekdays after both the Spring and Autumn clock changes, although this trend was more pronounced in Spring. Prior studies of the Spring transition have reported sleep duration on the following weekdays being up to 60 min lower (Barnes & Wagner, 2009; Lahti et al., 2006a; Medina et al., 2015; Völker et al., 2023; Zolfaghari et al., 2023), no different (Michelson, 2011), or up to 28 min higher (Tonetti et al., 2013) than the previous week. Likewise, studies have reported weekday sleep duration after the Autumn change being 25 min lower (Tonetti et al., 2013), no different (Barnes & Wagner, 2009; Michelson, 2011; Zolfaghari et al., 2023), or 2 min more (Jin & Ziebarth, 2020) than the previous week. Our estimates of weekday differences therefore are in line with previous studies. The fact that we find some weak evidence of catch up sleep after the Autumn clock change (when we gain an hour's sleep) suggests that the disruption to circadian rhythms caused by the clock change (rather than just lost sleep on the night of the clock change) could potentially be leading people to be more tired and sleep for longer.

Some research suggests that catching up on sleep can help people to recover from the effects of sleep loss on pain tolerance, mood, fatigue, cognitive performance and metabolic function (Dinges et al., 1997; Jang et al., 2023; Stroemel‐Scheder et al., 2020; Vgontzas et al., 2007). However, other studies have found that recovery sleep is insufficient to fully restore brain or metabolic function (Depner et al., 2019; Pejovic et al., 2013; Wu et al., 2006). How long it takes to recover from acute sleep loss is not entirely clear, with one study reporting it can take up to 4 days to recover from 1 hr of lost sleep (Kitamura et al., 2016), and another indicating that 3 nights of sleep are needed to make up for 1 night of insufficient sleep (Althoff et al., 2017). Future studies are needed to shed light on whether recovery sleep after the Spring clock change benefits health and, if so, how much recovery sleep is needed.

We did not find evidence that the effects of the DST clock changes on sleep on the Sunday of the clock change were modified by participant characteristics, with all groups experiencing an increase or decrease in sleep on the clock change Sunday compared with the previous and subsequent Sundays. However, we did detect some differences by sex, age and employment status when comparing sleep duration on the weekdays before and after the clock changes. In Spring (and to a smaller extent Autumn), women slept less on the weekdays after the clock change than before, whilst men slept more. This suggests that women are more vulnerable to sleep deprivation after the Spring transition. This may be because women generally have a higher prevalence of insomnia symptoms and diagnoses than men (de Lange et al., 2024), and these issues are exacerbated by the clock change, particularly in Spring when they lose sleep on the Sunday of the transition. Furthermore, our sample consisted of participants aged 43 years and over. It is therefore likely that many of the women in our study were perimenopausal or postmenopausal. As both of these stages are associated with higher levels of sleep disturbance (Nowakowski et al., 2013), this could make the women in our study particularly susceptible to the disruptive effects of the clock changes.

In Autumn, we found that those in the oldest age group and those who were retired slept less on the weekdays after the transition than before, whilst the youngest age group and those who were employed slept for longer. Logically, the sleep of older participants is more adversely affected by the Autumn clock change than those who are younger because as people age their chronotype shifts earlier, towards becoming a morning type (Druiven et al., 2021; Roenneberg et al., 2007). This means that when the clocks go back an hour in Autumn, older people may find it harder to adjust and lie in to their usual waking up time in the new time. However, this explanation is not supported by the fact that we did not find evidence of effect modification by chronotype in this study. It may be that older people are particularly vulnerable to their sleep being disrupted by the clock changes because sleep becomes lighter and more fragmented as we age (Lavoie et al., 2018). Nevertheless, this has important health implications as the resulting fatigue following the clock change could lead to an increase in falls and injuries in older people (Pana et al., 2021) or make “sundowning” (a deterioration in neuropsychiatric symptoms in the evening) worse in those with dementia (Canevelli et al., 2016). Further research is needed to identify the best ways to minimise clock change‐induced sleep loss in older adults.

Overall, our data indicate that the negative effects of the clock changes on sleep duration in Spring are more short‐lived than some previous studies suggest. Most importantly, we did not find evidence that the sleep loss associated with the Spring transition lasted for a whole week. This inconsistency with some previous research may be due to the larger sample size used in our study (*n* = 11,780), our use of objective actigraphy data rather than self‐report data, cultural differences or sample selection bias. In addition, this study only examined sleep duration. It is possible that the effect of the clock changes on the quality, efficiency and timing of our participants' sleep (which was beyond the scope of this study) may be longer. Nonetheless, our finding of increased sleep duration after the Spring clock change does correspond with an earlier study that reported sleep duration on Monday–Friday being 28 min higher than before, albeit in male university students (Tonetti et al., 2013).

An increase in sleep on the weekdays after the Spring clock change can be explained using the two‐process model of sleep regulation. After the clock change, sleep duration increases because time awake (and therefore sleep pressure) increases (Borbély, 1982). Essentially, losing sleep on the Sunday of the Spring clock change and then having to get up earlier during the following week makes people tired and so they sleep for longer. This process is then exacerbated by the misalignment between the body's internal biological clock (their natural circadian rhythm) and the social clock the person is now trying to live by. That said, the acute sleep loss observed in this study is not necessarily inconsequential for health, as research suggests that just 1 night of sleep loss (< 6 hr of sleep) is associated with an immediate decline in self‐reported mental health, as well as an increase in the number and severity of physical health symptoms (Lee, 2022). Furthermore, the clock changes themselves have been associated with an increase in cardiovascular events (Chudow et al., 2020; Jiddou et al., 2013; Sipilä et al., 2016), traffic accidents (Fritz et al., 2020) and depressive episodes (Hansen et al., 2017).

A strength of this study is the large sample size, aided by data from multiple years, which meant our overall estimates were more precise than those of previous studies. In addition, we had data for both Spring and Autumn clock changes, which enabled us to compare the effects of the transitions. Furthermore, this study benefited from objectively assessed sleep duration using wrist‐worn accelerometers. When tested against data from wearable cameras, time‐use information and sleep diaries, the machine learning model used to create the sleep data in this study correctly identified sleep with a very high level of accuracy: 91% of sleep was correctly identified (overall Kappa agreement score for all activity states: 0.69) (Doherty et al., 2018). That being said, the accelerometer‐measured sleep duration reported in this study is quite high for the age of the people studied, and is generally higher than the self‐reported sleep duration. It is possible that here the accelerometer and self‐report measures are suffering from measurement error in opposite directions. For example, sleep duration can be underestimated in self‐report data, particularly amongst those reporting a shorter sleep duration (Girschik et al., 2012), whilst actigraphy can overestimate time asleep by incorrectly recording inactivity whilst awake as sleep (Sadeh & Acebo, 2002).

Our study has several limitations. Unlike previous studies, participants did not have data for the whole study period. As a result, within‐individual comparisons were not possible. Secondly, the UK Biobank does not represent the wider UK population, with participants being older, more affluent and healthier than non‐participants (Fry et al., 2017). In addition, self‐reported lifestyle factors such as sleep duration, chronotype and employment status were collected at baseline so may not have been accurate when the participants wore the accelerometers several years later. Finally, our dataset only included information on sleep duration, not the timing of sleep, so it was outside the scope of the current study to explore the effects of the clock changes on circadian rhythms.

In conclusion, this study adds to the growing body of evidence that the shift forward to DST in Spring is associated with an acute loss of sleep. However, we found that this was more short‐lived than previous, smaller studies suggest. While sleep loss occurred on the Sunday of the Spring clock change, sleep duration on the weekdays following the transitions was not adversely affected. In fact, there was some evidence of catch up sleep after both clock changes. Future research should use large datasets with longer periods of accelerometer wear to capture sleep duration before and after the transition in the same individuals. It could also examine other aspects of sleep architecture such as circadian misalignment, sleep fragmentation, daytime napping/dozing or time asleep versus time awake.

## AUTHOR CONTRIBUTIONS

**Melanie A. de Lange:** Conceptualization; writing – original draft; writing – review and editing; formal analysis; project administration; methodology; investigation. **Rebecca C. Richmond:** Writing – review and editing; supervision. **Kate Birnie:** Writing – review and editing; supervision. **Chin Yang Shapland:** Writing – review and editing; supervision. **Kate Tilling:** Writing – review and editing; supervision. **Neil M. Davies:** Conceptualization; writing – review and editing; supervision; methodology.

## FUNDING INFORMATION

This research was funded in whole or in part by the Wellcome Trust (grant number 226909/Z/23/Z). For open access, the author has applied a CC BY public copyright licence to any Author Accepted Manuscript version arising from this submission. M.A.dL. is funded by the Wellcome Trust (grant number 226909/Z/23/Z). M.A.dL., R.C.R., K.T., K.B. and C.Y.S. work in a unit that receives support from the University of Bristol and the UK Medical Research Council (grant numbers MC_UU_00032/1, MC_UU_00032/02, MC/UU/00032/3, MC/UU/00032/4). N.M.D. is supported by the Norwegian Research Council (grant number 295989) and the UCL Division of Psychiatry (https://www.ucl.ac.uk/psychiatry/division-psychiatry). RCR is supported by Cancer Research UK (grant number C18281/A29019) and NIHR Oxford Health Biomedical Research Centre (grant number: NIHR203316).

## CONFLICT OF INTEREST STATEMENT

The authors have no conflicts of interest to declare.

## Supporting information


**DATA S1.** Supporting Information.

**FIGURES S1–S12**.

**TABLES S1–S8**.

## Data Availability

The UK Biobank dataset used to conduct the research in this paper is available via application directly to the UK Biobank. Applications are assessed to meet the required criteria for access, including legal and ethical standards. More information regarding data access can be found at https://www.ukbiobank.ac.uk/enable-your-research. Full code for all analyses is available at https://github.com/MeldeLange/dst_accel.
