# Supplementary material for: The effects of daylight saving time clock changes on accelerometer‐measured sleep duration in the UK Biobank
Source: J Sleep Res. 2024 Oct 21;34(3):e14335. doi: 10.1111/jsr.14335 (PMC12069741; doi:10.1111/jsr.14335)
Supplement: Supplementary file 2 — FIGURES S1–S12. [file JSR-34-e14335-s001.docx]

**Figures S1-S10** Mean daily sleep duration by day over the Spring and Autumn clock change fortnights, stratified by sociodemographics

**SPRING CLOCK CHANGE**

**Figure S1** Mean daily sleep duration by day over the Spring clock change by sex

**Figure S2** Mean daily sleep duration by day over the Spring clock change by age

**Figure S3** Mean daily sleep duration by day over the Spring clock change by employment status

**Figure S4** Mean daily sleep duration by day over the Spring clock change by habitual sleep duration

**Figure S5** Mean daily sleep duration by day over the Spring clock change by chronotype

**AUTUMN CLOCK CHANGE**

**Figure S6** Mean daily sleep duration by day over the Autumn clock change by sex

**Figure S7** Mean daily sleep duration by day over the Autumn clock change by age

**Figure S8** Mean daily sleep duration by day over the Autumn clock change by employment status

**Figure S9** Mean daily sleep duration by day over the Autumn clock change by habitual sleep duration

**Figure S10** Mean daily sleep duration by day over the Autumn clock change by chronotype

**SENSITIVITY ANALYSIS**

**Figure S11** Mean daily sleep duration by day over the Spring clock (excluding night shift workers)

**Figure S12** Mean daily sleep duration by day over the Autumn clock (excluding night shift workers)
